# Brewers’ Spent Grain: An Unprecedented Opportunity to Develop Sustainable Plant-Based Nutrition Ingredients Addressing Global Malnutrition Challenges

**DOI:** 10.1021/acs.jafc.3c02489

**Published:** 2023-07-10

**Authors:** Laura Nyhan, Aylin W. Sahin, Harold H. Schmitz, Justin B. Siegel, Elke K. Arendt

**Affiliations:** 1 School of Food and Nutritional Sciences, University College Cork, T12K8AF Cork, Ireland; 2 Graduate School of Management, University of California, Davis, Davis, California 95616, United States; 3 Genome Center, University of California, Davis, Davis, California 95616, United States; 4 Chemistry Department, University of California, Davis, Davis, California 95616, United States; 5 Department of Biochemistry and Molecular Medicine, University of California, Sacramento, Sacramento, California 96516, United States; 6 APC Microbiome Institute, University College Cork, T12YT20 Cork, Ireland

**Keywords:** Brewers’ spent grain, BSG, upcycling, sustainability, life cycle assessment, malnutrition, world hunger, food waste

## Abstract

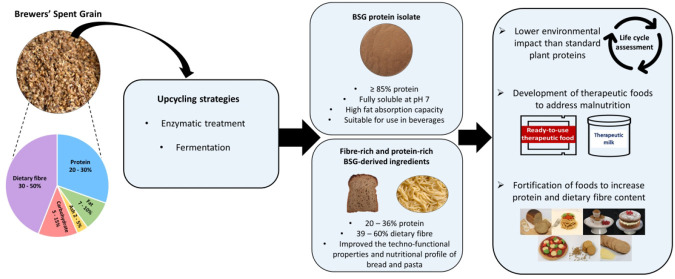

There is an urgent requirement to minimize food waste and create more sustainable food systems that address global increases in malnutrition and hunger. The nutritional value of brewers’ spent grain (BSG) makes it attractive for upcycling into value-added ingredients rich in protein and fiber having a lower environmental impact than comparable plant-based ingredients. BSG is predictably available in large quantities globally and can therefore play a role in addressing hunger in the developing world via the fortification of humanitarian food aid products. Moreover, addition of BSG-derived ingredients can improve the nutritional profile of foods commonly consumed in more developed regions, which may aid in reducing the prevalence of dietary-related disease and mortality. Challenges facing the widespread utilization of upcycled BSG ingredients include regulatory status, variability of raw material composition, and consumer perception as low-value waste products; however, the rapidly growing upcycled food market suggests increasing consumer acceptability and opportunities for significant market growth via effective new product innovation and communication strategies.

## Introduction

Food insecurity is a significant global issue with an estimated 345.2 million people expected to be food insecure in 2023, an increase of 200 million since 2020.^1^ Moreover, the 2022 Global Hunger Index indicates that progress to address world hunger is at a standstill, with approximately 828 million people suffering from undernourishment globally in 2021.^2^ This figure is expected to increase in the coming years, with current projections showing that the world is not on track to reach the United Nations Sustainable Development Goal (SDG) 2 Zero Hunger by 2030 unless profound changes are made. One such change is the reduction in food loss and waste (FLW), a global problem which results in an estimated one-third of all food produced for human consumption lost each year, amounting to around UDS $936 billion.^3^ 14% is lost after harvesting and prior to reaching retailers, with a further 17% wasted in retail and by consumers. Moreover, this is predicted to increase in the coming years as a result of population and economic growth.^4^ FLW is detrimental to the environment, accounting for an estimated one-quarter of the land, water resources, and fertilizers used globally while also contributing to greenhouse gas emissions, loss of biodiversity, and air and water pollution.^5^ Such events have contributed to a rise in the global average temperature, leading to global warming and subsequent impacts on sea levels and precipitation, which in turn can cause extreme weather events such as drought or flooding. The depletion of natural resources and climate change events can disproportionately affect areas which rely on the agricultural sector for livelihood, particularly in developing nations which already suffer from poverty and food insecurity.^6^

As defined by the Upcycled Food Association, “upcycled foods use ingredients that otherwise would not have gone to human consumption, are procured and produced using verifiable supply chains, and have a positive impact on the environment”.^7^ The production of upcycled foods aligns with SDG 12 Responsible Consumption and Production and, in particular, target 12.3, to halve global food loss and waste along the food supply and production chains by 2023. Upcycling of food processing side streams and byproducts has gained attention in recent years within the framework of a circular economy, with the aim of minimizing food waste and aiding in the transformation to more sustainable food systems.

Brewers’ spent grain (BSG), the insoluble solid residue of malted barley, is the most abundant byproduct of the brewing process, representing 85% of the total brewing waste material produced. With BSG obtained at approximately 20 kg/hL of beer brewed, around 36.4 million tonnes of BSG is generated globally per annum.^8^ BSG has a short shelf life due to its high moisture content and susceptibility to microbial spoilage, with current outputs mainly restricted to low-value animal feed or landfill.^9^ Such practices are unsustainable, with the supply of BSG often exceeding the feed demands of local farmers, and each tonne of BSG in landfill releasing approximately 513 kg CO_2_ equivalent of greenhouse gases.^10^ BSG can be dried to extend its shelf life and facilitate its use as a food ingredient, and while it is a promising source of human nutrition due to its dietary fiber and protein content, the inclusion of BSG in food systems can negatively affect the technofunctional and sensorial characteristics of the products.^11−14^ Thus, the implementation of processing strategies such as enzymatic treatment or fermentation may be beneficial in enhancing the functional performance of BSG as a food ingredient.

This review will provide an overview of the composition of BSG, while outlining how upcycling strategies, such as enzymatic hydrolysis and fermentation, can improve the performance of BSG in food systems. A summary of BSG-containing foods currently available on the market will be provided, while the nutritional value, technofunctional properties, and food-based applications of commercial BSG-derived functional ingredients will be described. Although exploration of the economic viability of the production and implementation of BSG-derived ingredients is beyond the scope of this Review, the environmental impact of the production of BSG-derived ingredients in comparison to standard plant- and cereal-based ingredients will be described. Insight into the potential use of upcycled BSG ingredients to address malnutrition in the developing world and the Western diet will be given, while, finally, the opportunities and challenges for the implementation of upcycled BSG food ingredients will be discussed.

## BSG: A Nutrient-Rich Brewing Byproduct

**Figure 1:**
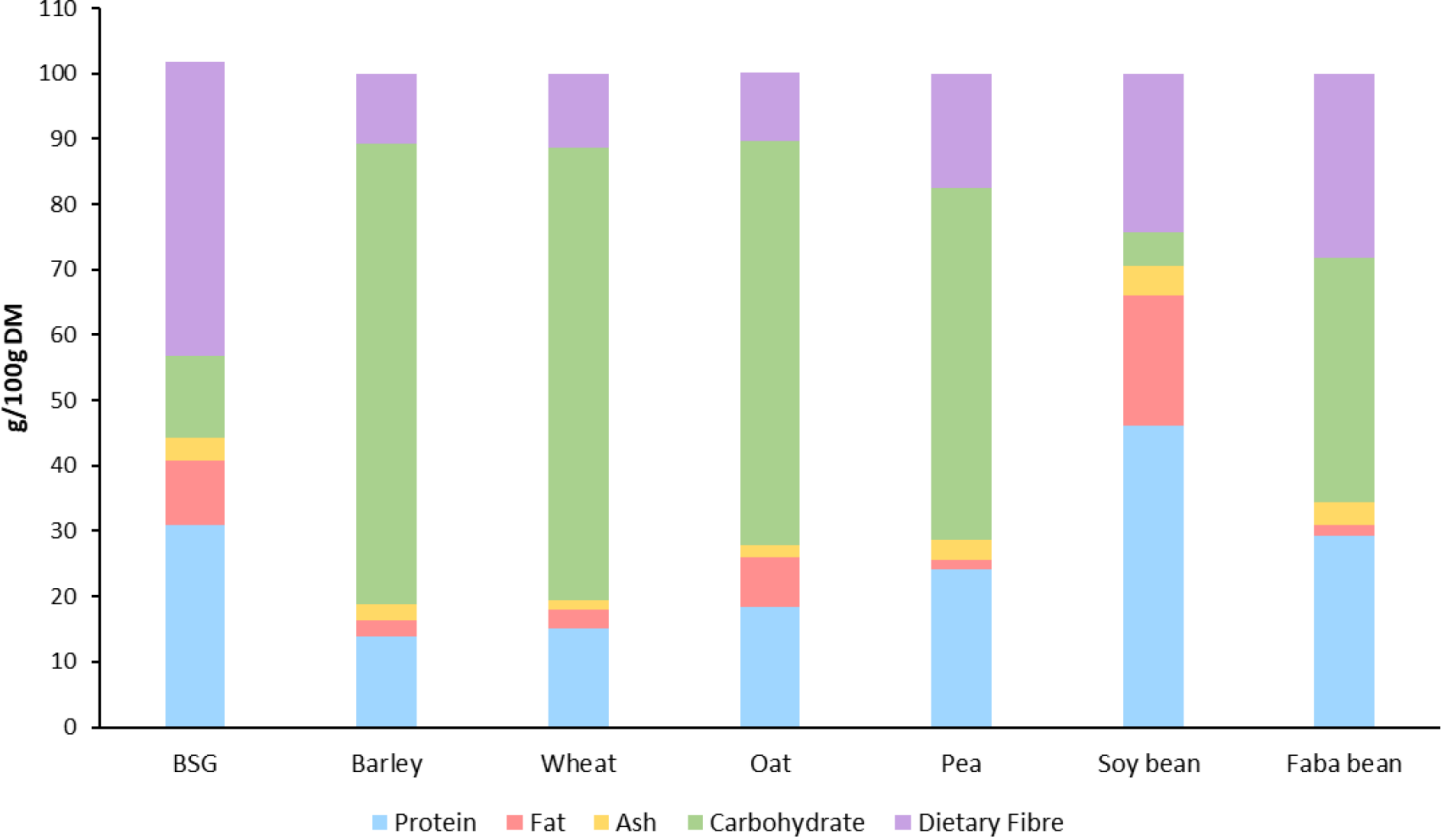
Proximate composition of BSG, unmalted barley, wheat, oat, pea, soy bean, and faba bean.^15,20−22,25,26^

The major components of BSG are the walls of the husk–pericarp–seed layers which surrounded the original barley grain and, depending on the efficiency of the mashing regime, varying levels of starchy endosperm and the walls of empty aleurone cells.^9^ The composition of BSG can vary considerably due to differences in barley variety, harvest time, hop type, malting and mashing processes, and the absence or presence of adjuncts during the brewing process.^9^ However, regardless of intrabrewery variations in chemical composition, BSG is considered a lignocellulosic material rich in both protein and fiber. To compare, the composition of BSG alongside unmalted barley and some common cereal and legume sources is shown in Figure 1; though it should be noted that this is a comparison between a byproduct whose nutritional value has been enhanced by processing and unprocessed cereal and legume raw materials. As most of the starch present in the barley grains is removed during the brewing process, the dietary fiber content of BSG can range from 40 to 50%,^11,15−17^ significantly higher than that of unmalted barley, wheat, and oat (9–20%)^11,18−21^ and legume sources including pea (14–21%),^22,23^ soy bean (21–25%),^24,25^ and faba bean (11–28%)^22,26,27^ The main fiber constituent in BSG is hemicellulose which comprises 20–40% of the total composition.^15,28^ The hemicellulose fraction of BSG primarily consists of arabinoxylan, a dietary fiber which has been linked with potential health benefits such as prebiotic activity, improved glycemic control, and antioxidant activity.^15,29−31^ Cellulose and lignin are the other abundant polysaccharides in BSG, whose contents can range from 16 to 29%,^28,32,33^ and 12 to 28%, respectively.^28,32−34^ β-glucan is also present in low levels, normally in the range of ∼1% w/w.^9^

BSG also has a relatively high protein content, typically around 30% on a dry weight basis.^15,35^ In comparison, this is approximately 2-fold higher than the protein content of unprocessed barley or wheat and almost 70% higher than that of oat (Figure 1). Although the protein content of BSG is lower than that of soy (33–50%)^25,36^ considering that other well-known raw legume sources such as pea, chickpea, lentil, and faba bean contain between 16 and 29% protein on a dry weight basis,^22,26,37,38^ BSG can be considered an equivalent, or in some cases, a superior protein source in comparison to commonly consumed cereals and legumes. With regard to amino acid composition, essential amino acids can represent up to 38% of the total protein content in BSG, a typical value for cereal proteins and similar to that of legume sources (Table 1). As is the case with most cereals, lysine remains the limiting amino acid in BSG, although the byproduct provides a considerable amount (up to 87%) of the recommended lysine requirement per gram of protein as outlined by the WHO/FAO/UNU 2007 report.^39^ In terms of protein quality, the digestible indispensable amino acid score (DIAAS) of BSG is not well documented in the literature, although a commercial barley–rice protein isolate derived from BSG reports a calculated value of 51%,^40^ similar to that of barley (51–55%) and wheat (43–56%).^41−43^ The DIAAS of protein sources such as pea and faba bean are within the range of 64–76%, while soy protein is considered high quality with DIAAS values of 98–103% reported.^43,44^ As legume proteins are typically high in lysine but deficient in methionine and cysteine, a combination with a complementary protein source such as BSG could serve to increase DIAAS and provide higher quality protein.

**Table 1:** Amino Acid Composition of BSG, Barley, Wheat, Oat, Pea, Soybean, and Faba Bean Protein^a^

	BSG^200^	Barley^201^	Wheat^202^	Oat^202^	Pea^84^	Soy^84^	Faba bean^22^
Essential Amino Acids (EAA)							
histidine	2.6	2.0	2.0	2.5	2.7	2.5	3.2
isoleucine	4.1	3.3	4.1	3.4	4.9	3.5	3.6
leucine	10.0	6.0	6.6	7.1	9.1	7.5	7.1
lysine	3.9	3.2	2.9	3.3	7.9	6.0	5.5
methionine	2.1	1.5	1.5	2.1	0.7	1.1	0.9
cysteine	b	2.1	1.8	1.7	c	c	1.1
phenylalanine	5.9	5.2	5.3	4.9	6.0	5.3	4.3
threonine	3.7	3.2	3.0	3.2	3.9	3.7	3.4
tryptophan	b	1.7	b	b	0.5	0.8	b
valine	5.2	4.5	4.6	4.6	5.5	3.8	3.9
∑EAA	37.5	32.7	31.8	32.8	41.2	34.2	33.0
Nonessential Amino Acids (NEAA)							
alanine	6.1	3.3	3.8	3.9	4.5	4.0	3.7
arginine	5.2	4.5	5.3	7.3	10.4	8.2	7.7
aspartic acid	7.0	5.4	5.7	6.6	11.4	10.1	10.2
glutamic acid	21.2	22.6	23.8	19.4	17.9	17.4	14.2
proline	10.2	10.0	10.4	5.8	5.0	5.1	b
serine	4.5	4.1	5.1	4.0	5.8	5.5	4.3
tyrosine	3.9	2.8	2.2	2.7	4.1	3.9	2.7
glycine	3.6	3.2	4.2	4.4	4.7	4.1	3.4
∑NEAA	61.7	55.9	60.5	54.1	63.8	58.3	46.2

a Values expressed as % total protein.

b Not measured.

c <LOQ, values were below the limit of quantification.

Compared to cereals, BSG has a relatively high fat content of 7–10%, with essential fatty acids comprising over half of total BSG lipids.^11^ BSG also contains minerals, of which the most abundant are magnesium, calcium, and phosphorus. In addition, BSG retains many of the bioactive components found in whole barley, such as ferulic, *p*-coumaric, sinapic, syringic, and caffeic acids,^45−47^ phenolic compounds which may potentially demonstrate antioxidant, anticarcinogenic, and immunomodulatory activity.^9,48^

## Strategies for Upcycling BSG

In its simplest and most common form, upcycling of BSG consists of drying the ingredient, often followed by milling and possible sieving and subsequent incorporation into a food product. To date, BSG has mainly been used in bakery products including bread, breadsticks, muffins, cookies, and pizza dough, and some nonbakery applications such as pasta, yogurt, frankfurters, and sausages.^11−14,49−57^ BSG inclusion can significantly enhance the nutritional quality of foods, even with relatively low addition levels. For example, Czubaszek et al. partially replaced wheat flour with BSG (10%), resulting in a bread with twice as much dietary fiber than the control, while increasing the BSG inclusion level to 20% resulted in a further 50% increase in the dietary fiber content.^53^ However, while nutritionally beneficial, the impact of BSG inclusion on the quality and sensorial characteristics of food must also be considered. In bread, the cutoff point for BSG inclusion appears to be ∼10% with levels exceeding this leading to increased density, reduced specific volume, and decreased consumer acceptability.^11,12,14^ A similar scenario can be observed for pasta, whereby Nocente et al. reasoned that replacement of 10% flour by BSG is a good compromise between increased protein and dietary fiber content and acceptable technological and sensory characteristics.^13,55^ So, while it is theoretically possible to elevate the nutritional profile of foods significantly through the addition of high levels of BSG, realistically this is possible only up to a certain point, after which the nutritional benefits are outweighed by the negative quality characteristics. Further processing strategies may be employed in order to increase the functionality and technological performance of BSG in food systems, of which the most common are enzyme treatment and fermentation technology. An overview of these processing methods and their application to BSG will be provided in the following sections; however, it should be noted that the economic viability and sustainability of implementing these upcycling strategies at an industrial scale was not considered, as this was beyond the scope of the review.

### Enzymatic Treatment

Enzymatic hydrolysis allows the cleavage of long biopolymers into their smaller units, a process that can be exploited to solubilize BSG components for improved accessibility. Although the costs associated with commercial enzymes can be high, enzymatic treatment is considered a more sustainable approach than chemical extraction methods, mainly because the use of toxic solvents such as methanol, diethyl ether, n-hexane, and ethyl acetate is not required.^33,58^ In addition, the application of enzymatically extracted compounds in food products is viewed more favorably than the incorporation of those extracted by chemical means.^9^ Enzymatic hydrolysis of BSG can be tailored to obtain specific components, with studies utilizing carbohydrases, proteases, and esterases to obtain products such as monosaccharides, cellulose- and hemicellulose-derived oligosaccharides, solubilized arabinoxylan, peptide-rich fractions, and also phenolic compounds such as ferulic acids which are bound to the lignocellulosic structure of BSG.^9,59^ Of interest to the food industry is the ability of hydrolysis to increase the functionality of BSG, with Vieira et al. producing a BSG protein hydrolysate that possessed increased antioxidant activity and significantly improved emulsifying characteristics.^60^ Similiarly, Celus et al. generated BSG protein hydrolysates which showed increased solubility at acidic and alkali pH values, increased emulsion-forming capacity, comparable or improved foaming capacity, and improved foaming stability.^61^ However, not all hydrolysates demonstrated such an improvement in technofunctional characteristics, with the type of enzyme used and the degree of hydrolysis majorly impacting the properties of the resulting hydrolysates.^61^ The efficacy of enzymatic treatment as an upcycling tool for BSG becomes particularly evident when considering the application of BSG hydrolysates in food matrices, with examples outlined in Table 2. Cermeño et al. found that the inclusion of enzymatically hydrolyzed BSG (BSGB) positively affected the viscoelastic properties of muffin batter and produced muffins which had a higher height and softer texture than those containing unmodified BSG (BSGA),^62^ with the authors hypothesizing that the improved technofunctional properties of BSGB was likely due to the solubilization of carbohydrates and protein and subsequent interactions with other macromolecules. Moreover, it was found that BSGB could be incorporated at a level up to 10% without negatively affecting the appearance or texture likeability, while the unmodified BSG was only tolerated at a maximum level of 5%. It should be noted that although the dietary fiber content of the muffins containing BSGB was significantly higher than that of the control muffins (not containing BSG), they contained 34–46% less fiber than those containing unmodified BSG due to the probable hydrolysis of BSG polysaccharides by carbohydrases during enzyme treatment. Despite this, the use of enzymatically modified BSG remains justifiable due to its ability to strike a balance between enhanced nutrition and improved technological performance.^62^ BSG hydrolysates have also been applied in a bread system, increasing the fiber content from 2.82 g/100 g (refined wheat flour) to 6.9 g/100 g (20% w/w fermented BSG (fBSG) substitution) and allowing for a “high fiber” health claim to be made. The release of bioactive compounds from BSG during hydrolysis was also apparent, with the fBSG bread possessing almost 2-fold more total polyphenols than the refined bread control.^63^ Naibaho et al. demonstrated the potential of BSG hydrolysates as fat replacers in nondairy yogurt, observing a less dense fat network and distribution in coconut-based yogurt containing various BSG protein hydrolysates (BSGPs).^64^ The authors had also demonstrated the enhanced antioxidant activity of these BSGPs in a previous study;^65^ however it was not investigated whether this improved activity translated to the yogurt product, a concept which would have been interesting to explore.

**Table 2:** Processing of BSG and Application in Food Products

upcycling strategy	process conditions	food product	proportion of BSG incorporated	main finding(s)	ref
enzyme treatment and fermentation	hydrolysis with an enzyme mixture for 12–24 h at 55 °C followed by fermentation with *Lactiplantibacillus plantarum* F10 and/or *Lacticaseibacillus rhamnosus* LGG for 8–24 h at 25–27 °C (patent no. WO 2018/033521 A1)	bread	4–18% of baker’s flour replaced with BSG or fermented BSG (fBSG) (w/w) to reach “source of fiber” (3 g/100 g fiber) and “high fiber” (6 g/100 g fiber) claims	inclusion of fBSG in bread resulted in increased specific volume, reduced crumb hardness, increased microbial shelf life, and a slower release of reducing sugars over time during in vitro starch digestion compared to control BSG	(35)
enzyme treatment and fermentation	hydrolysis with an enzyme mixture for 12–24 h at 55 °C followed by fermentation with *Lactiplantibacillus plantarum* F10 and/or *Lacticaseibacillus rhamnosus* LGG for 8–24 h at 25–27 °C (patent no. WO 2018/033521 A1)	pasta	2.00–14.96% of flour replaced with BSG or fermented BSG (fBSG) (w/w) to reach “source of fiber” (3 g/100 g fiber) and “high fiber” (6 g/100 g fiber) claims	inclusion of fBSG reduced the glycemic index of pasta compared to control BSG	(17)
fermentation	10 g of ground, autoclaved BSG mixed with 50 mL of sterile water, inoculated with 10^6^ CFU/g of *Bacillus subtilis* WX-17 and fermented at 37 °C for 72 h; samples were filtered, and the supernatant was collected and analyzed	beverage	16.7% (w/v)	fermentation produced a nutritious beverage (liquid phase of fermentation) with increased levels of 13 amino acids, higher antioxidant content, and increased phenolic compounds	(79)
fermentation and protein extraction	BSG fermented with *Rhizopus* anisospores (10^7^ spores/mL) at 37 °C for 3 days, freeze-dried, ground into powder and sieved (sieve size 400 μm); proteins extracted by ethanolic-alkali extraction	mayonnaise	BSG protein (BSGP) and fermented BSG protein (fBSGP) mixed with whole egg and added to mayonnaise formulation (10%); ratio of BSGP or fBSGP to whole egg: 40% (f)BSGP + 60% whole egg; 60% (f)BSGP + 40% whole egg; 100% (f)BSGP	fBSGP displayed superior emulsifying stability, water/oil binding capacity, foaming capacity, and antioxidant activity to BSGP; fBGSP demonstrated improved emulsion stability in terms of creaming, microstructure, and viscosity in mayonnaise	(68)
fermentation	UHT skim milk supplemented with BSG flour, inoculated with *Streptococcus thermophilus* TH-4 and *Lacticaseibacillus paracasei* subsp. *paracasei* F-19 and fermented at 37 °C until pH 5.4 was reached	fermented milk	1% (w/v)	BSG did not influence fermentation kinetics or microbial population numbers, but enhanced the survival of *S. thermophilus* TH-4 against *in vitro* simulated gastrointestinal stress	(72)
fermentation	BSG filtered and liquid fraction (100 μm) collected (LBSG), mixed with a commercial soy drink (SoD) and fermented with strains of *Lactiplantibacillus plantarum* and *Lactococcus lactis* at 30 °C for 18 h	yogurt	20:80 LBSG to SoD (% v/v)	inclusion of 20% LBSG resulted in a product with a protein content, acidity level, and texture/sensory characteristics similar to that of a dairy yogurt	(71)
enzyme treatment and fermentation	ground BSG homogenized with water at a 60:40 ratio, supplemented with xylanase (Depol 761P, 100 nanokatal (nkat)/g) and incubated at 50 °C for 5 h; enzyme-treated BSG inoculated with 10^7.5^ CFU/g of *L. plantarum* PU1 and incubated at 37 °C for 24 h	pasta	15% of semolina (w/w) replaced with BSG or fermented BSG (fBSG)	compared to control BSG, the use of enzyme treated and fermented BSG resulted in pasta with higher protein digestibility and quality indices (biological value, protein efficiency ratio, essential amino acid index, and nutritional index), improved technological and sensorial characteristics, and increased antioxidant activity	(70)
fermentation and enzyme extraction	BSG adjusted to 55% moisture (w/w) with water, inoculated with 10^7^ spores/g of *Aspergillus awamori* IOC-3914 and incubated at 30 °C for 96 h (air water saturation of 90%)	bread	20% of wheat bran (w/w) replaced with fermented BSG (48 h) and 14% of water (v/v) replaced with crude enzymatic extract of fermented BSG	bread containing fermented BSG contained 198% more soluble ferulic acid than control bread, but had a decreased volume and higher density	(69)
fermentation	moisture content of substrate (stale sourdough breadcrumbs mixed with BSG) adjusted to 40% with distilled water, inoculated with 2.7 × 10^6^ and 1.4 × 10^6^ of *Neurospora intermedia* CBS 131.92 and *Rhizopus oryzae* CCUG 28,958, respectively, and incubated at 35 °C for 6 days	stale sourdough bread	0–20% (w/w)	fermentation of stale sourdough bread mixed with 6.5% or 11.8% BSG by *N. intermedia* or *R. oryzae* resulted in a product with textural properties similar to a commercial soybean burger	(80)
enzyme treatment	sheared and pH-adjusted BSG incubated with 75 μL g^–1^ BSG dry weight of each carbohydrase (Biocellulase A, Bioglucanase FS2000 and Bioglucanase HAB) at 50 °C for 1 h; pH of the suspension was adjusted to pH 9.3 and suspension was incubated at 50 °C for 2 h with Alcalase 2.4 L (2%, v/w, BSG protein) followed by the addition of Bioprotease FV (1% v/w, BSG protein) and incubation for 2 h at 50 °C	muffins	5, 10, 15% (w/w)	inclusion of enzyme-treated BSG resulted in muffins with higher height, darker color, and decreased hardness compared to the control	(62)
fermentation	BSG substituted with sucrose (10% w/w), inoculated with 10^6^ CFU/g of *Weissella confusa* A16 and incubated at 25 °C for 24 h	bread	33% (w/w)	presence of dextran and maltosyl-isomaltooligosaccharides along with the increased protein and fiber levels in breads containing fermented BSG resulted in higher free amino acid bioaccessability and a positive effect on gut microbiota functionality	(76)
fermentation	BSG mixed with milk, pasteurized at 90 °C for 15 min, cooled to 38–43 °C, inoculated with 0.05% (w/w) of microbial culture (*Streptococcus thermophilus*, *Lactobacillus delbreuckii* subsp. *bulgaricus*, *Lactobacillus acidophilus*, and *Bifidobacterium lactis*) and incubated at 43 °C until a pH of 4.3–4.8 was reached	yogurt	BSG mixed with milk at ratios of 0:100, 5:95, 10:90, 15:85, and 20:80 (BSG/milk; w/w),	inclusion of BSG decreased the fermentation time, maintained the flow behavior and stability of the yogurt, and supported the survival of LAB during the 14 days chilled storage	(56)
enzyme treatment	BSG mixed with water at a ratio of 1:10, incubated without protease treatment (BSGP-C), with 0.5% Protamex (BSGP-P), or with 0.5% Protamex and 0.1% Flavourzyme (BSGP-PF) at 50 °C for 3 h and heated to 90 °C for enzyme inactivation	coconut-based yogurt	BSG mixed with water-soluble coconut extract (WSCE) at a ratio of 1:9 (w/w)	use of BSG derivatives resulted in yogurt alternatives with a less dense fat distribution, a more homogeneous matrix, and a 3-fold higher lactic acid concentration	(64)
enzyme treatment	BSG treated with protease (Alcalase) and carbohydrase (Cellulclast 1.5 L at two levels (0 and 0.1% w/w) according to a central composite design to produce BSG flour (FBSG)	bread	20% of wheat flour (w/w) replaced with FBSG	FBSG bread had a higher fiber content, total polyphenol content, and antioxidant activity than the control bread, but a decrease in specific volume and an increase in rubberiness was observed	(63)
fiber extraction	washed, defatted BSG gelatinized with 0.6% Termamyl at 95 °C for 1 h, washed four times with hot distilled water (100 °C) and cooled at room temperature; residues were washed with 99.9% ethanol (60 °C), filtered, and dried	chicken patties	1, 2, 3, 4% of BSG dietary fiber extract (w/w)	addition of 3% BSG dietary fiber extract resulted in the lowest cooking loss, no significant difference in protein solubility, no change in patty diameter and the highest sensorial acceptability	(81)
fiber extraction	washed, defatted BSG gelatinized with 0.6% Termamyl at 95 °C for 1 h, washed four times with hot distilled water (100 °C) and cooled at room temperature; residues were washed with 99.9% ethanol (60 °C), filtered, and dried; BSG pre-emulsion prepared by combining carboxymethyl cellulose, ice, and BSG dietary fiber extract and homogenizing for 5 min	chicken sausages	20, 25, 30% of pork fat replaced with BSG pre-emulsion (w/w)	inclusion of BSG pre-emulsion improved the hardness, chewiness, and gumminess of the reduced-fat chicken sausages, while having no influence on cohesiveness; addition of BSG pre-emulsion up to 25% had no significant difference in acceptability of the chicken sausages	(49)

### Fermentation

Fermentation is a valuable upcycling tool that can enhance the safety, sensory, functional, and nutritional characteristics of foods and ingredients. As outlined by Zeko-Pivač et al., the suitability of BSG as an ideal raw material for fermentation has been exploited for the production of high-value products such as enzymes, organic acids, xylitol, and volatile fatty acids, to name but a few.^8^ However, of particular relevance to this review is the application of fermentation as an aid to improve the nutritional and functional performance of BSG in food products, a concept that this section will explore in more detail.

Fungal fermentation can significantly enhance the nutritional value of BSG, mainly through an increase in the crude protein content and amino acid levels. Zeko-Pivač et al. and Eliopoulos et al. reported increases of 11–50% in the protein contents of BSG samples which underwent solid state fermentation by the fungi *Trametes versicolor* and *Pleurotus ostreatus*, respectively,^10,66^ while another study demonstrated that *Rhizopus* fermentation significantly increased the protein content of BSG from 20.5 g/100 g to 31.7 g/100 g.^67^ Although the effect of fungal fermentation on the nutritional profile of BSG has been investigated extensively, studies whereby the upcycled ingredient is incorporated into a food matrix are limited (Table 2). Chin et al. performed solid-state fermentation of BSG with *Rhizopus oligosporus* and subjected the residue to ethanolic–alkali extraction to produce protein hydrolysates. As the hydrolysates exhibited superior emulsifying, foaming, and water/oil binding abilities, the authors investigated their use as plant-based emulsifiers in mayonnaise. The results were promising, with the fermented hydrolysate demonstrating better emulsion stability than the unfermented hydrolysate with regard to creaming, viscosity, and microstructure.^68^ The application of *Aspergillus awamori*-fermented BSG and its crude enzymatic extract in bread resulted in a product with 198% more ferulic acid than the control; however, the bread was noted to have a decreased specific volume and a denser crumb. The authors hypothesized that this was likely due to the high xylanase, amylase, and protease activities of the BSG fermentate and corresponding enzymatic extract causing the dough to lose its air retention capacity.^69^ It is also plausible that the replacement of 20% wheat bran by fermented BSG was too high an inclusion level, with the impact of excessive BSG content on the technological aspects of bakery products previously discussed and well-documented. It would be of interest to apply the BSG fermentate and the corresponding crude enzymatic extract singly to examine the individual effects of the byproducts on the technological properties of the bread.

Fermentation of BSG is not limited to fungal species; lactic acid bacteria (LAB) are also commonly utilized. In contrast to fungi, fermentation of BSG by LAB is often preceded by enzymatic hydrolysis to increase the availability and accessibility of nutrients due to the fastidious nature of the microbes.^9^ Neylon et al. integrated BSG and a hydrolyzed, fermented BSG (fBSG) into bread at varying levels to achieve “source of fiber” and “high fiber” claims according to EU regulations and investigated the effect on dough and bread quality and nutritional value.^35^ Although the inclusion of both BSG ingredients resulted in a decreased specific volume compared to the control bread, bread produced with fBSG exhibited a higher specific volume and reduced crumb hardness than those containing unfermented BSG.^35^ A follow-on study was performed that investigated the impact of the same BSG ingredients in pasta; it was found that fBSG inclusion at a high addition level (HF) resulted in a product with a lower predicted glycemic index than observed with BSG inclusion (HF). This may have been due to the higher amount of resistant starch present in fermented BSG or a reduction in starch bioavailability due to the promotion of interactions between starch and gluten by lactic acid.^17^ Schettino et al. also reported that the inclusion of 15% fermented BSG in pasta resulted in a lower predicted glycemic index than the addition of unfermented BSG, despite the comparable carbohydrate contents of the samples. Interestingly, this study investigated the effect of fermented BSG addition on protein quality indices (digestibility, essential amino acid profile, biological value, protein efficiency ratio, and nutritional index). Although the protein levels of the control BSG pasta (BSG-p) and the fermented BSG pasta (fBSG-p) did not differ significantly, fBSG-p was characterized as having a higher *in vitro* protein digestibility (+16%) than BSG-p, along with significantly higher chemical scores for several essential amino acids. Determination of the nutritional index (NI), a global predictor of protein quality that considers both qualitative and quantitative indicators, showed that fBSG-p (2.5) had an almost 2-fold higher value than BSG-p (1.3). Such nutritional improvements are commonly associated with LAB-fermented foods due to the occurrence of proteolysis during fermentation and a subsequent increase in small peptides and free amino acids. The pasta fortified with bioprocessed BSG decreased the generation of reactive oxygen species (ROS) in human colon carcinoma cells (Caco-2), even after simulated in vitro gastric digestion.^70^

An alternative approach to the two-step process of BSG fermentation and subsequent application in a food product is the use of BSG as a substrate in foods that undergo fermentation, e.g., yogurts and fermented beverages. Naibaho et al. found that the inclusion of BSG (5–20%) in a yogurt system significantly increased the fermentation rate, did not negatively affect LAB growth during 14 days refrigerated storage, and reduced the level of syneresis.^56^ A separate study also investigated the use of BSG in a yogurt system but instead used the liquid fraction of filtered BSG in combination with a commercial soy drink as the fermentation substrate. The final product was similar to a commercial product in terms of acidity and protein content and displayed a firm structure before stirring and a thinner, smoother structure after stirring. The authors speculated that the differences in texture before and after stirring could in fact result in the production of two different products from a single fermentation, further improving the efficiency of the process.^71^ An interesting study by Battistini et al. demonstrated that the inclusion of 1% BSG (w/v) in the fermentation of UHT milk by *S. thermophilus* TH4 and *L. paracasei* F-19 conferred a significant improvement in the survival of TH4 against simulated in vitro gastrointestinal stress, highlighting the potential of BSG as a prebiotic ingredient in yogurt production.^72^

Fermented foods have been shown to have a positive impact on the microorganisms (bacteria, archaea, eukarya) colonizing the gastrointestinal tract (gut microbiota) due to either the bioactive compounds produced during fermentation or interactions with microbes from the fermented food which survive the gastrointestinal tract.^73^ BSG and its constituents (arabinoxylan, lignin) have been shown to have a modulatory effect on the human gut microbiota, promoting the growth of beneficial bacteria such as *Bifidobacterium* spp. and *Lactobacillus* spp. and stimulating short-chain fatty acid (SCFA) production.^15,29,74,75^ However, work investigating the impact of fermentation on the microbiota modulatory effect of BSG is scarce, with few studies published on this topic. Koirala et al. fermented sucrose-supplemented BSG with *Weissella confusa* A16 to induce the synthesis of dextran and maltosyl-isomaltooligosaccharide exopolysaccharide (EPS) and investigated its use as an ingredient in wheat bread.^76^ Simulated *in vitro* digestion of EPS-positive BSG bread (EPS+BB) and EPS-negative BSG bread (EPS–BB) using Simulator of Human Intestinal Microbial Ecosystem (SHIME) and fecal metabolite analysis showed that both fermented BSG breads had a significant effect on gut microbiota, positively influencing SCFA and free amino acid (FAA) metabolism after 1 week of treatment. In particular, the presence of dextran and maltosyl-isomaltooligosaccharides in EPS+BB bread increased FAA bioaccessibility and decreased ammonia production. However, as control breads which did not contain BSG or which contained unfermented BSG were not included in the study, the observed effects cannot be attributed solely to the fermentation process. Nevertheless, the positive impact of the fermented BSG breads on gut health is significant. Although both BSG breads demonstrated gut modulatory capacity, it was found that EPS+BB had a higher specific volume, significantly lower hardness values, and reduced staling rate compared to EPS–BB, functional improvements characteristic of dextran inclusion in bread.^77,78^

## Commercial Upcycled BSG Products

**Figure 2:**
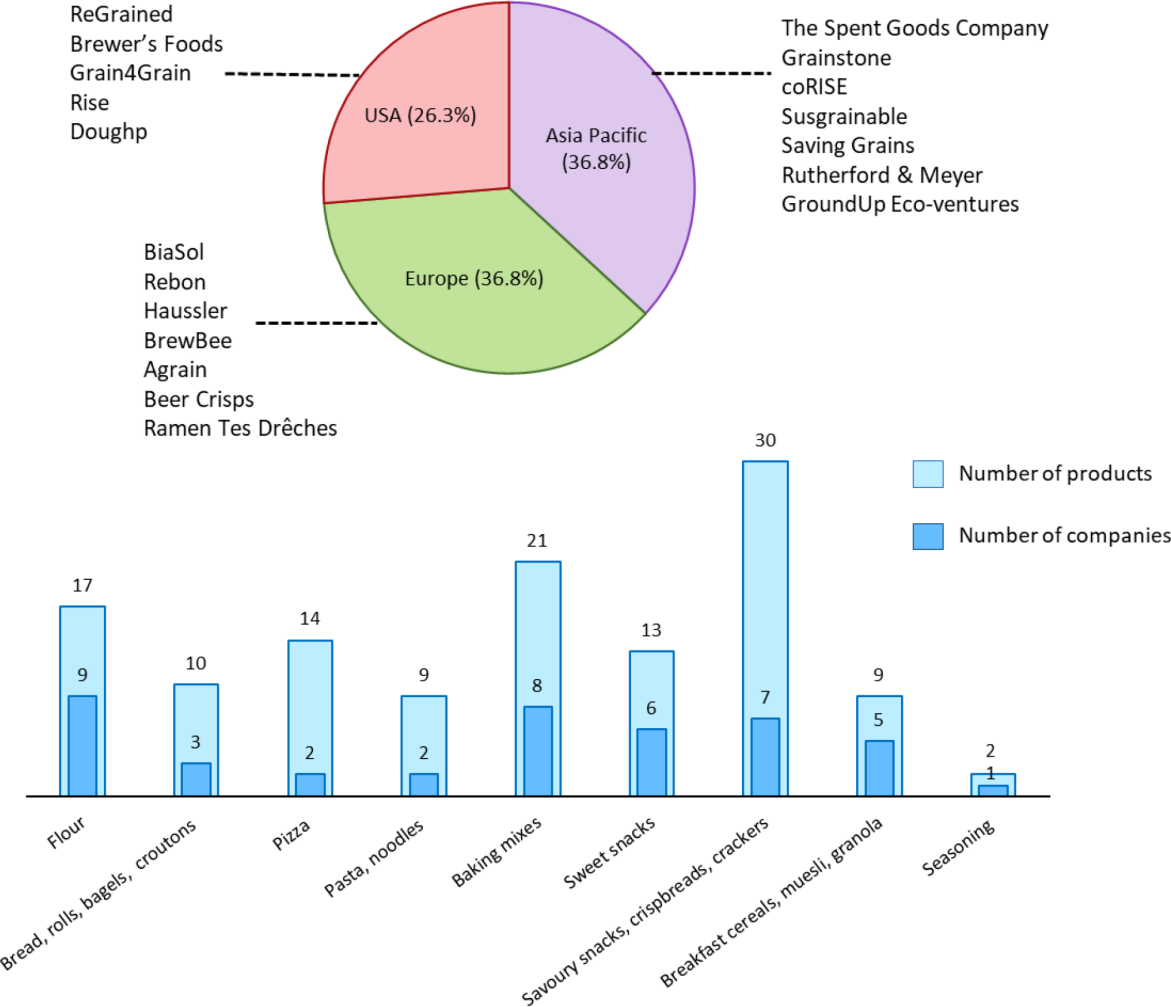
Companies utilizing upcycled BSG as a food ingredient and categories of associated food products.

With the demand for dried spent grain forecast to reach a valuation of USD 24 billion by the end of 2033,^82^ the number of companies utilizing spent grain as a food ingredient is on the rise. A market analysis identified 19 food manufacturers operating in the US, Europe (Ireland, France, Germany, Denmark, Switzerland, UK) and Asia Pacific (New Zealand, Australia, Canada, India) which incorporate dried BSG into food products (Figure 2). Of these, two were Upcycled Certified (ReGrained and Grain4Grain). A total of 125 products were identified, the majority of which were savory snacks, crackers, and flatbreads (24%). Savory snacks comprised chips and snack puffs from companies including ReGrained, Brewer’s Foods, Rutherford & Meyer, and Brewbee, all available in a variety of flavors. 42% of companies surveyed produced baking mixes for products such as brownies, banana bread, pancakes, and carrot cake, with such mixes accounting for 17% of all products analyzed. Considering the abundance of studies in the literature regarding the functionality of BSG in pasta, the lack of this product commercially is surprising, with just ReGrained producing an upcycled BSG pasta. Bread products and pizzas made up 8% and 11% of products, respectively, with the high amount of pizza products attributed mainly to BrewBee who produce a variety of pizzas with BSG-fortified bases. Sweet snacks (cereal bars, brownies, cookies) and breakfast cereals, muesli, and granola comprised 10% and 7% of all products, respectively. Nine companies (BiaSol, Rise, Grainstone, coRISE, Grain4Grain, Saving Grains, Ramen Tes Drêches, GroundUp Eco-ventures, and Susgrainable) offer an upcycled BSG flour, with the fiber and protein contents of these products ranging from 30 to 46% and 18 to 30%, respectively, similar to values found in the literature. 84% of companies analyzed provide a range of different product types, with BrewBee offering the widest variety (27% of total products analyzed), including pizza, muesli, breakfast cereal, panettone, and savory snacks (Tschipps and Trellini). The use of BSG in a seasoning mix is rare with Grain4Grain the only company using the ingredient in this manner. Of all the products surveyed, just one was certified as low FODMAP by Monash University - the Premium Brewers Flour produced by Grainstone.

## Commercial BSG-Derived Ingredients

**Figure 3:**
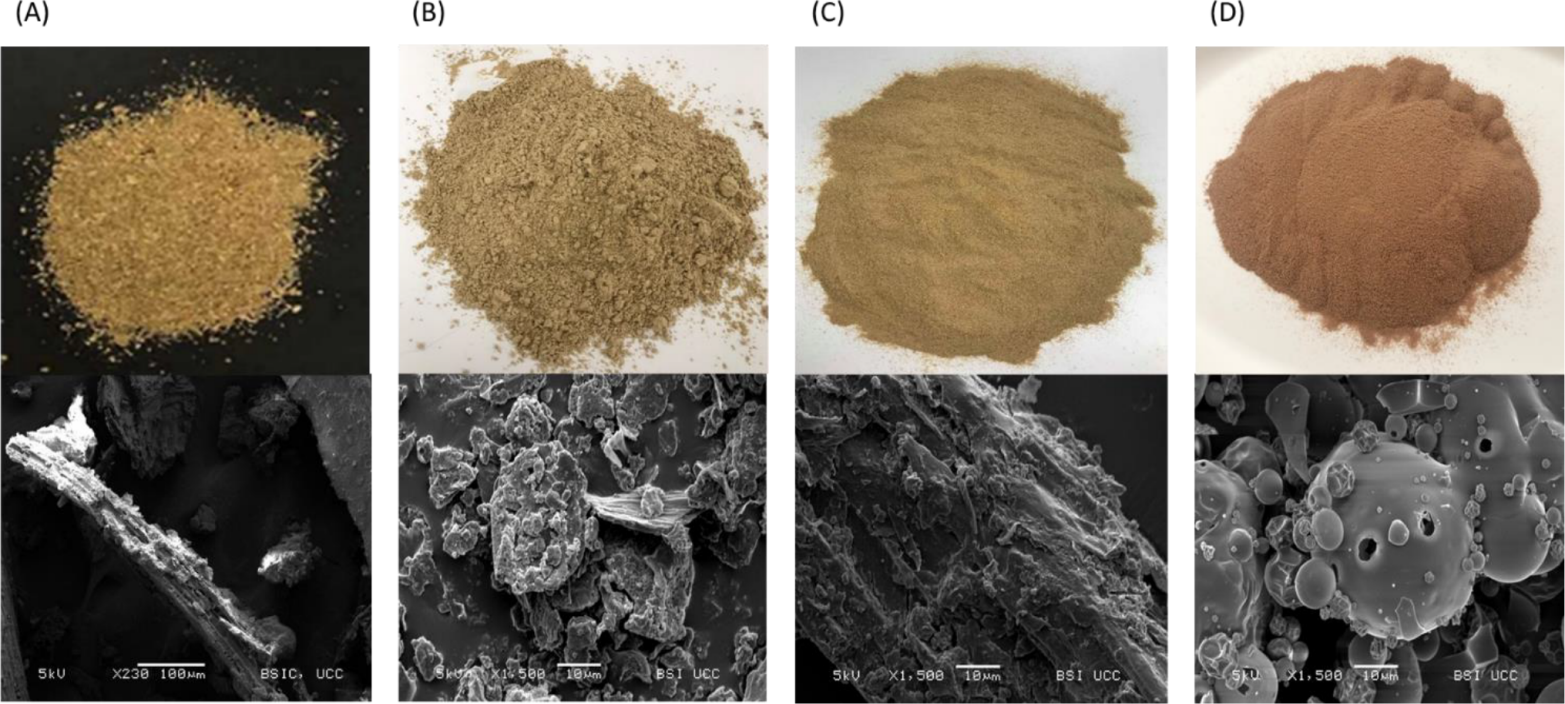
Photographic (top) and scanning electron micrograph images (bottom) of BSG (A) at 230× and fiber-rich BSG-derived ingredient (B), protein-rich BSG-derived ingredient (C), and BSG protein isolate (D) at 1500× magnification.

Aside from incorporating BSG into food products, companies are also recognizing and seizing the opportunity to produce and provide functional BSG-derived ingredients to the food industry at commercial scale. Sustainable Ingredients, a Swiss company, offers a barley protein ingredient (45% protein), a barley fiber ingredient (70% fiber), and a barley flour (26% protein, 45% fiber) to food manufacturers for suggested use in products such as cookies, crispbreads, yogurts, pizza, pasta, cereals, and baked goods. Alongside their food product offerings, the aforementioned US company ReGrained also acts as a wholesale ingredient supplier, providing the upcycled BSG ingredient SuperGrain+ to food businesses for new product innovation. Moreover, the company has also partnered with Puratos to offer SuperGrain+ Sourdough Systems for use in bakery applications, while a collaboration with Kerry Group has resulted in the development of SuperGrain+ Protein Crisps as an ingredient for the development of snack bars and bites. The Swiss company Circular Food Solutions recently added Legria powder to their portfolio, an upcycled BSG ingredient containing 20% protein and 54% fiber which is available to purchase directly, with plans also in place to start producing plant-based meat alternatives using the ingredient by mid-2023. EverGrain, a subsidiary of Anheuser Busch InBev, produces three BSG-derived ingredients: a BSG protein isolate (EverPro), a BSG-derived protein-rich ingredient (EverVita Prima), and a BSG-derived fiber-rich ingredient (EverVita Fibra). The BSG protein isolate is the first ingredient of its kind on the market; as the BSG used in this isolate production is obtained from a brewing process which uses rice as an adjunct to barley, the ingredient is considered a barley–rice protein isolate. The appearance and microstructure of BSG and BSG-derived ingredients are shown in Figure 3. The fibrous structures of BSG can be seen, along with starch granules, which appear to be embedded in the matrix (Figure 3A). The BSG-derived protein-rich ingredient (Figure 3B) is represented by small fibrous compounds (fiber) and small particles on the surface (protein particles and starch granules), while the BSG-derived fiber-rich ingredient (Figure 3C) includes elongated fibrous compounds with starch granules and protein particles also visible.^83^ As previously reported by Jaeger et al., the BSG protein isolate (Figure 3D) displays round particles that vary greatly in size, likely due to the combination of both barley and rice proteins in the matrix. The presence of holes on the surface of the particles indicates damage, which could be attributed to the processing methods used in the production of the isolate.^84^

### Nutritional Value of BSG-Derived Ingredients

The composition of the BSG-derived ingredients in comparison to BSG is given in Table 3. BSG-derived protein-rich ingredient has a protein content of 36 g/100 g, slightly higher than that of BSG (31 g/100 g). Although the fiber-rich BSG-derived ingredient contains less protein than the others, the ingredient can still be considered a relatively good protein source, providing 21 g of protein per 100 g. In comparison, the BSG protein isolate contains significantly more protein (87%) than BSG or the BSG-derived ingredients, with the reported protein content within the range of what has been determined previously for this ingredient, as well as for commercial isolates derived from pea and soy (81–89% protein).^84,85^ The amino acid compositions of the BSG-derived ingredients are similar to each other and to that of BSG, with essential amino acids comprising 36–42% of the total protein content. Naturally, lysine is the limiting amino acid in each of the BSG-derived ingredients; however, the ingredients still provide a significant amount (82–91%) of the required lysine amount per gram of protein as outlined by the WHO. A similar scenario was reported by Jaeger et al., who determined that BSG protein isolate provided 81% of the recommended daily lysine requirement per gram of protein, while also highlighting the inability of both pea and soy protein isolates to meet the sulfur-containing amino acid requirements.^84^ Elsewhere, Sahin et al. found that the concentrations of amino acids in the protein-rich and fiber-rich BSG-derived ingredients were up to 75% higher than in baker’s flour or wholemeal flour.^86^ The study also investigated the protein profile of the ingredients, demonstrating that while both ingredients contained the same protein fractions, the BSG-derived fiber-rich ingredient showed thicker bands in the 15–28 kDa range, indicating an increased presence of those specific fractions. The authors concluded that this may be attributed to enhanced protein extraction as a result of the higher ash content of the ingredient or due to fiber preventing the formation of protein complexes. Analysis of the protein profile of BSG protein isolate by Jaeger et al. demonstrated the presence of small proteins (4–15 kDa), with undefined bands indicating protein degradation and the presence of peptides with varying molecular weights.^84^

**Table 3:** Nutritional Composition of BSG and BSG-Derived Ingredients

	BSG^a^	BSG-derived protein-rich ingredient^b^	BSG-derived fiber-rich ingredient^b^	BSG protein isolate^c^
Proximate Composition (g/100 g DM)				
protein	31.0	36.0	21.0	87.0
fat	9.7	12.0	9.0	0.8
saturated	2.7	3.3	2.1	0.2
trans	<0.4	0.0	0.0	0.0
carbohydrates	12.6	10.0	7.0	3.9
sugars	0.4	1.0	1.0	0.5
dietary fiber	44.8	39.0	60.0	3.1
cellulose	d	16.7	22.4	d
hemicellulose	d	19.1	30.5	d
lignin	d	3.2	7.1	d
ash	3.6	3.0	4.0	5.5
Essential Amino Acids (g/100 g protein)				
histidine	2.6	2.2	2.6	2.1
isoleucine	4.1	3.9	4.0	3.2
leucine	10.0	8.9	10.0	7.4
lysine	3.9	3.9	4.1	3.7
methionine	2.1	2.0	2.3	2.0
cysteine + cystine	d	1.9	2.1	1.3
phenylalanine	5.9	5.6	5.6	5.9
threonine	3.7	3.8	4.0	4.2
tryptophan	d	1.4	1.4	1.4
valine	5.2	5.6	5.7	4.8
Nonessential Amino Acids (g/100 g protein)				
alanine	6.1	5.2	6.2	5.1
arginine	5.2	5.5	5.8	5.7
aspartic acid	7.0	6.8	7.0	9.3
glutamic acid	21.2	20.9	19.7	24.3
proline	10.2	10.5	7.1	9.6
serine	4.5	4.7	4.7	5.0
tyrosine	3.9	3.4	3.7	4.1
glycine	3.6	3.9	4.1	4.4

a Data sourced from Lynch et al. (2021)^15^ and Nazzaro et al. (2021).^200^

b Fiber-rich and protein-rich BSG-derived ingredients produced by drying, milling, and air-classification of BSG. Data was obtained from ingredient specifications provided by the manufacturer.

c BSG protein isolate produced by enzymatic hydrolysis of BSG, followed by purification, filtration, and spray-drying. Proximate composition data of BSG protein isolate was obtained from the ingredient specification provided by the manufacturer. Amino acid composition of BSG protein isolate sourced from Jaeger et al. (2023).^84^

d Not determined.

The BSG-derived fiber-rich ingredient has an exceptionally high dietary fiber content of 60 g/100 g, which is 34% higher than that of unprocessed BSG. Despite the BSG-derived protein-rich ingredient containing less dietary fiber (39%), the ingredient can still be considered a rich source of fiber. In contrast, BSG protein isolate contains just 3.1 g/100 g dietary fiber, a low fiber content which is characteristic of protein isolate ingredients which typically contain <5% dietary fiber.^87−89^ BSG protein isolate contains much less fat (0.8%) than BSG, or the protein-rich and fiber-rich BSG-derived ingredients, likely due to the reduction of lipid compounds during processing. The trace amount of fat in BSG protein isolate in comparison to other protein isolates which can contain up to 8.5% lipids^84,89^ is beneficial, reducing the potential for oxidation and subsequent negative impacts on flavor, texture, and color.^90^ The ash contents of the protein-rich and fiber-rich BSG-derived ingredients (3–4%) are similar to that of BSG (3.6%) and also to values published previously for these ingredients (3.1–4.3%),^86,91^ while a slightly higher ash value was determined for BSG protein isolate (5.5%).

### Applications of Protein-Rich and Fiber-Rich BSG-Derived Ingredients

To date, the impact of the protein-rich and fiber-rich BSG-derived ingredients on the technofunctional properties and nutritional profile of foods has been investigated in bread and pasta. Sahin et al. incorporated the ingredients into bread at varying inclusion levels to reach “source of fiber” (SF) and “high fiber” (HF) nutrition claims and investigated the subsequent impact on dough and bread quality and nutritional values.^86^ The addition of the fiber-rich BSG-derived ingredient resulted in bread (SF) with a specific volume comparable to that of the baker’s flour control. Increasing the addition level of the ingredient (HF) led to a slight decrease in volume; however, this was still significantly higher than the wholemeal flour control bread. The addition also ameliorated hardness in comparison to the control bread and did not significantly impact the crumb structure. The inclusion of the protein-rich BSG-derived ingredient (SF) did not negatively impact specific volume; however, the higher addition level (HF) resulted in a decreased specific volume along with a harder crumb. Nevertheless, the technological performance of the BSG-derived ingredients in bread was superior to that of dried BSG, as Neylon et al. observed that the inclusion of BSG to reach a HF claim resulted in bread with a lower specific volume and higher hardness value.^35^ As previously discussed, studies have reported a significant deterioration in bread quality when the addition level of BSG exceeds 10%; thus, the addition of up to 11–16% of the BSG-derived ingredients while maintaining an acceptable bread quality is noteworthy. Moreover, Neylon et al. also reported that the inclusion of BSG at any addition level had no effect on the microbial shelf life of bread,^35^ whereas it was found that the incorporation of BSG-derived ingredients resulted in an extended shelf life with the first microbial growth observed after 9–9.3 days instead of 6–6.7 days (control breads). With regard to nutritional value, the replacement of flour with the ingredients at HF levels increased the protein content of bread by up to 36%, while having no negative impact on the amino acid score. Of significance was the predicted increase in the lysine concentration (+24.5%) of the bread fortified with the protein-rich BSG-derived ingredient (HF), while tryptophan was expected to comprise 0.22%-0.56% of the protein in breads containing the BSG-derived ingredients. Considered a limiting amino acid in cereal-based products, tryptophan was not predicted to be present in the control breads, highlighting the potential of BSG and BSG-derived ingredients to act as natural tryptophan fortifiers and elevate protein quality.

The ability of the same BSG-derived ingredients to enhance the nutritional value of pasta has also been demonstrated. Cuomo et al. determined that the inclusion of 15% of the protein-rich BSG-derived ingredient resulted in pasta which contained 15–20% more protein (17.8–18.1 g/100 g) and 174–181% (8.5–8.7 g/100 g) more fiber than the semolina control, achieving “high protein” (HP) and HF nutrition claims.^91^ To compare, others have reported lower pasta protein contents of 13–15 g/100 g with the inclusion of 15–20% BSG.^13,70^ Neylon et al. also incorporated BSG into pasta at a similar level (15%), reaching a fiber value of 6 g/100g,^17^ lower than what was achieved through the addition of an equivalent amount of protein-rich BSG-derived ingredient (8.5–8.7 g/100 g).^91^ Moreover, Sahin et al. reported a protein content of 7.8 g/100 g and a fiber level of 3.2 g/100 g in pasta fortified with just 1% of the protein-rich BSG-derived ingredient.^83^ The potential of the fiber-rich BSG-derived ingredient as a dietary fiber source in pasta has also been demonstrated, with fiber contents of 6.3–7.9 g/100 g determined for pasta containing 9.5–10% of the ingredient,^83,91^ while in another study, almost 2-fold more BSG was required to reach a comparable dietary fiber level.^13^ Similar to observations made by Sahin et al.,^86^ the use of BSG-derived ingredients in pasta also enhanced protein quality.^91^ Pasta formulated with the replacement of semolina by 15% of the protein-rich BSG-derived ingredient or 10% of the fiber-rich BSG-derived ingredient demonstrated chemical scores of 52 and 46, respectively, higher than that of the semolina control (43). While lysine remained the limiting amino acid in all formulations, the increase in the chemical score of pasta fortified with BSG-derived ingredients highlights the improvement in the biological value of the protein.^91^

### Technofunctional Properties of BSG Protein Isolate

Knowledge of the physicochemical and technofunctional properties of protein ingredients is essential for their application in food products. The functional characteristics of commercially available products such as pea and soy protein isolates have been well documented; however, few studies have focused on BSG protein. Jaeger et al. recently investigated the technofunctional properties of the BSG protein isolate in comparison to pea protein isolate (PPI) and soy protein isolate (SPI), evaluating solubility, foaming characteristics, surface hydrophobicity, zeta potential, emulsifying properties, and rheological behavior.^84^ The results from the study are summarized in Table 4, in addition to data associated with other plant proteins for comparison purposes. BSG protein isolate was found to have a relatively small particle size, approximately 11.8-fold, 5.8-fold, and 2.7-fold smaller than SPI, PPI, and lentil protein isolate (LPI), respectively, and similar to that of blue lupin protein isolate (BLPI). As is clear from Table 4, the particle size of protein isolates has been found to vary greatly, with higher values indicative of poorly dispersed, large particles remaining present in solution, an undesirable trait for food applications.^87,88^ The BSG protein isolate (1% protein, w/w) was found to be fully soluble (101.71%) in water at neutral pH, likely due to the increased presence of small peptides and amino acids as a result of protein degradation during brewing, while the small particle size of the isolate in the dispersion was likely an additional contributing factor. With the protein solubility of isolates produced from sources such as soy, pea, faba bean, lentil, and lupin reported to be in the range of 9–76.9%,^84,85,87,89,92,93^ the superior solubility of the BSG protein isolate is a distinct advantage for the food industry for which the low solubility of plant protein isolates is a significant challenge. Protein solubility is closely associated with zeta potential, a value which can provide insight into the behavior of a particle in solution. The zeta potential of the BSG protein isolate was comparable to that of BLPI and white lupin protein isolate (WLPI); however, the BSG protein isolate was found to be almost 25–32% more soluble than these isolates; thus, the higher degree of solubility is likely a result of the aforementioned protein degradation. A similar observation was made for SPI which had a similar zeta potential to BSG protein isolate but a 2-fold less degree of solubility. Most studies investigating the protein solubility and zeta potential of protein ingredients do so across a range of pH values to assess the suitability of the ingredient for use in acidic or alkaline food applications. While BSG protein isolate displays exceptional protein solubility at pH 7, further work investigating the effect of pH would be beneficial for food manufacturers.

**Table 4:** Technofunctional Properties of Protein Isolates Derived from BSG, Soy, Pea, Faba Bean, and Lentil

	particle size *D*[4,3] (μm)	protein solubility at pH 7 (%)	zeta potential at pH 7 (mV)	fat absorption capacity (%)	foaming capacity (%)	foaming stability (%)	surface hydrophobicity	ref
BSG protein isolate	12.22	101.71	–30.03	182.35	112.68	45.57	a	(84)
pea protein isolate	70.48	22.27	–22.60	157.72	38.19	80.12	4292.20	(84)
soy protein isolate	144.33	51.96	–33.80	120.05	70.14	74.31	7471.40	(84)
faba bean protein isolate	b	9.49	b	65.32	18.06	70.00	2183.00	(92)
lentil protein isolate	32.80	43.00	∼−20	224.00	33.30	15.90	2411.00	(88)
white lupin protein isolate	51.50	69.80	∼−30	b	∼60	>85	842	(87)
blue lupin protein isolate	12.10	76.90	∼−30	b	∼60	>90	2185	(87)

a Not applicable.

b Not mentioned.

BSG protein isolate had the highest foaming capacity (112.68%) but a low foaming stability, while the opposite was true for PPI and faba bean protein isolate (FPI). The foaming properties of proteins are known to be closely interlinked with other physicochemical properties such as protein solubility and surface hydrophobicity and are also highly dependent on factors such as protein concentration, pH, temperature, extraction process, and foaming method.^94^ It should also be noted that the foaming properties of BSG protein isolate, PPI, SPI, and FPI were determined using 2% sample dispersions, whereas the values for LPI, BLPI, and WLPI are representative of 1% protein dispersions; thus, the values are not entirely comparable. The high foaming capacity and low foaming stability of BSG proteins have previously been documented, with Connolly et al. reporting a foaming capacity of 1177% for a BSG protein isolate at pH 12, but a maximum foaming stability of just 29%. In addition, the foaming characteristics were significantly influenced by pH, with poor foaming properties observed at pH ≤ 8.^95^ It is of interest to measure the foaming properties of the BSG protein isolate at a range of protein concentrations and different pH values. The fat absorption capacity of proteins is a function that is important when considering application in high-fat food matrices such as dairy products, sauces, and bakery products. BSG protein isolate was characterized as having the highest fat absorption capacity in comparison to PPI, SPI, or FPI, surpassed only by LPI (Table 4). BSG protein hydrolysates have previously been reported to have fat absorption capacities of ca. 200–300%, with the high values attributed to the presence of small peptides and amino acids as a result of proteolysis, exposing more hydrophobic regions to the oil interface.^65,68^ Jaeger et al. also investigated the rheological properties of BSG protein isolate, demonstrating that the viscosity of dispersions remained unchanged during heating and cooling cycles, highlighting the potential for application in food matrices where gel formation is undesirable, e.g., plant-based beverages. In contrast, the occurrence of heat-induced gelation of legume proteins derived from pea, soy, lentil, lupin, and chickpea are well-documented in the literature,^84,85,87,88^ deeming these ingredients more suited for bakery products and yogurt and cheese alternatives.

## Environmental Impact of BSG-Derived Ingredients

Food systems are sustainable if they generate positive value across three dimensions: economic, social, and environmental. The impact of food ingredients or products on the environmental dimension is determined by life cycle assessment (LCA).^96^ Generally, the upcycling of food waste is considered to have a positive impact not only on the environment^97,98^ but also on food security through the positive impact on food availability, addressing one of the four pillars of food security (accessibility, availability, stability, utilization).^99^ Several studies on the rejuvenation of different types of food waste, particularly vegetables and fruits,^97^ reveal a positive result for the environmental dimension of sustainability. However, as the knowledge of life cycle assessment grows, the methods and key indicators need to be carefully chosen.

**Figure 4:**
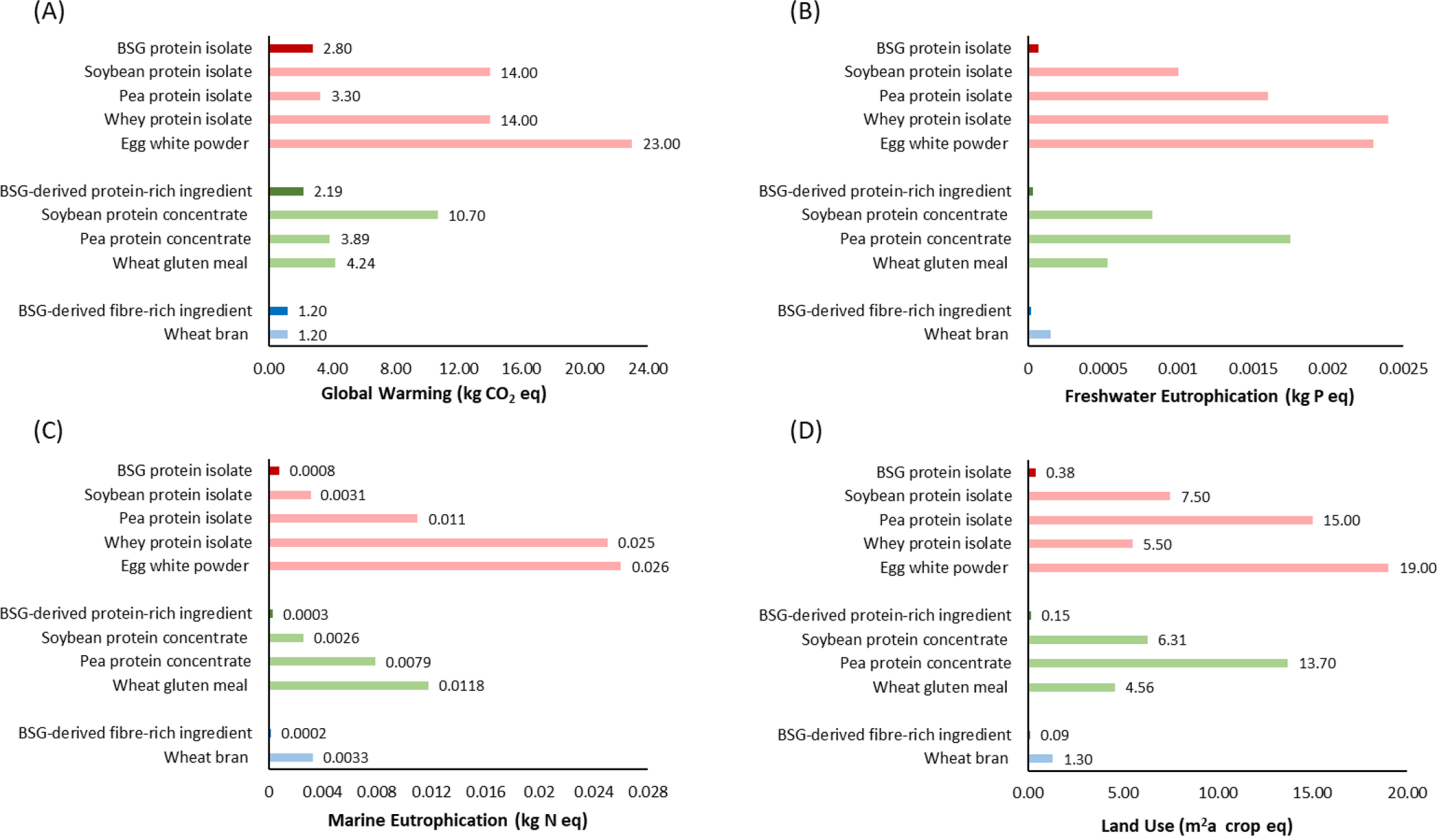
Global warming potential (A), freshwater eutrophication (B), marine eutrophication (C), and land use (D) for BSG protein isolate (EverPro), BSG-derived protein-rich ingredient (EverVita Prima) and BSG-derived fiber-rich ingredient (EverVita Fibra) in comparison to conventional food ingredients. Values were determined on the basis of production of 1 kg of protein (BSG protein isolate, BSG-derived protein-rich ingredient, soybean protein isolate and protein concentrate, pea protein isolate and protein concentrate, wheat gluten meal, and egg white powder) or 1 kg of fiber (BSG-derived fiber-rich ingredient and wheat bran).

Traditionally, BSG is used as landfill material and/or animal feed. However, these waste management strategies result in a high contribution to greenhouse gas emissions, particularly methane.^100^ Recent studies investigate the valorization of BSG to produce biofuels or packaging materials.^101,102^ However, the research in these areas is at a very early stage, and LCA has not been included in these studies. On the contrary, the use of BSG as an ingredient in food products has been investigated thoroughly, and LCA of BSG-rich snacks, for example, was determined to have a significantly lower impact on global warming compared to its use as feed or landfill.^100^ A more in-depth LCA of BSG-derived ingredients, which are used for human nutrition, was conducted by Blonk Consultants in 2021 following the ISO 14040 and 14044 LCA methodological standards.^103,104^ The BSG-derived ingredients of interest were a BSG protein isolate (EverPro), BSG ingredient rich in protein (EverVita Prima; 36% protein and 39% dietary fiber), and BSG ingredient rich in dietary fiber (EverVita Fibra; 21% protein and 60% dietary fiber). One part of the conducted LCA compares BSG protein isolate with the most common protein ingredients used in food, such as soy protein isolate, pea protein isolate, whey protein isolate, and egg white powder. The BSG protein isolate caused significantly lower global warming compared with all other protein ingredients (Figure 4), particularly animal-derived protein and soy protein isolate. The higher global warming impact of soybean protein isolate in comparison to pea protein isolate is likely due to emissions from land-use change for soybean production, a process which can contribute majorly to CO_2_ emissions.^105^ Eutrophication is of great concern since it can lead to the deterioration of water quality and the depletion of dissolved oxygen in water bodies.^106^ The generation of traditional protein ingredients showed a high impact on freshwater eutrophication and marine eutrophication, while the BSG protein isolate minimally impacted this environmental factor. Whey protein isolate had a high impact on eutrophication mainly due to the use of fertilizer and management of manure in the dairy farm, while the significant impact of egg white powder on freshwater and marine eutrophication can be attributed to feed and laying emissions during egg production.^107,108^ Furthermore, land use contributes to resource depletion, a factor which is urged to be reduced in the future.^106^ The generation of BSG protein isolate requires minimal land use compared to other plant-based or animal-based protein ingredients, which is putatively due to the fact that no additional farmland is required to produce the protein isolate as the land is already being used for barley cultivation, with the protein isolate produced from a byproduct of barley utilization in brewing. Overall, BSG protein isolate can be considered more sustainable regarding environmental impact compared with conventional protein ingredients used in food production. A second LCA part compared a BSG-derived ingredient rich in protein (EverVita Prima) with protein concentrates, which have a protein content between 40% and 60% (Figure 4). The most common protein concentrates used in food products are soy protein concentrate, pea protein concentrate, and wheat gluten meal. The analysis of the impact of the ingredients on global warming revealed very similar results for pea and wheat gluten meal, which were significantly lower than soy. The BSG-derived protein-rich ingredient showed the lowest global warming potential. The eutrophication impact of the BSG-derived protein-rich ingredient was reported to be 8–51 times less than that of the comparison ingredients. Wheat gluten meal had the highest impact on marine eutrophication of the ingredients investigated but conversely had a lower impact on freshwater eutrophication than soybean and pea protein concentrate. The eutrophication values for wheat gluten meal are within the range of those reported by Deng et al., who surmised that the use of pesticides and fertilizers was the primary contributor to marine eutrophication during wheat gluten production.^109^ The third and final part of the LCA compares a BSG-derived ingredient rich in dietary fiber (EverVita Fibra) with wheat bran. Wheat bran generally has a low environmental impact and does not differ from the BSG-derived fiber-rich ingredient regarding global warming. Although wheat bran undergoes less processing than the BSG-derived ingredient, it has a higher impact on cultivation and a net similar global warming impact. Compared to wheat bran, the BSG-derived fiber-rich ingredient showed a significantly lower impact on eutrophication. Nitrogen and phosphate emissions from fertilizer use were identified as the primary source of eutrophication during the production of wheat, in line with previously reported observations by Deng et al.^109^

## Upcycled BSG and World Hunger

### Malnutrition in Sub-Saharan Africa

Malnutrition is a significant global health problem, affecting almost one-third of the population, and is considered the leading cause of illness worldwide. According to the FAO, approximately 768 million people globally were estimated to be undernourished in 2020, with Sub-Saharan Africa accounting for 264 million (34.5%) of this cohort, the highest prevalence anywhere worldwide.^110,111^ Undernutrition is a significant contributor to child mortality, with nutrition during the first 1000 days of life having a profound impact on physical and mental development.^112^ Physiological indicators of undernourishment in children include stunting, wasting, and being underweight.^112^ Despite a decrease in the prevalence of stunting in children under the age of five in Africa in recent years, a significant proportion (30.7%) are still affected by the condition.^113^ The prevalence of wasting among children in sub-Saharan Africa (6%) in 2020 was below the global average (6.7%); however figures vary greatly between regions (3.2–6.9%).^113^ Poverty is a primary cause of hunger in Africa, with an estimated 490 million households surviving on less than $1.90 a day in 2021, a situation which was exacerbated by the COVID-19 pandemic.^114^ The cycle of poverty is difficult to escape, with children exposed to long-term undernourishment often suffering from long-term medical conditions which reduce labor productivity and earning potential.^115^ Conflict is another significant driver of hunger in Africa, with regions such as Ethiopia and South Sudan which are directly affected by conflict experiencing the most severe food insecurity through diminished employment and income-earning opportunities, increased pressure on food supply systems, and destruction of resources.^116^ Other factors which contribute to undernourishment and hunger are overpopulation, poor governance and corruption, and environmental challenges.^117^

### Therapeutic Foods for Treating Malnutrition

The treatment of severe acute malnutrition (SAM) can be achieved through the use of therapeutic foods, nutrient-dense products that are staples of humanitarian aid programs. Treatment with therapeutic milk (TM) feeds F75 and F100 is recommended for children who require hospital-based intervention for the most severe form of malnutrition, while ready-to-use therapeutic food (RUTF) can be used for community-based treatment. Until recently the guidelines for the formulation of RUTF stated that at least 50% of the protein should be derived from dairy sources; however in 2022 the Codex Alimentarius Commission adopted new guidelines on RUTF with protein quality now of greater importance than protein type, stipulating that cereals, legumes, seeds, or any other locally available ingredients can be used together with/instead of dairy protein, as long as a protein digestibility corrected amino acid score (PDCAAS) of ≥0.9 is achieved.^118^ Efforts are ongoing to reformulate RUTF with alternative protein sources, with several published studies and clinical trials investigating the use of legumes and cereals such as soy, oat, rice, sesame, chickpea, sorghum, and maize.^119−123^

**Figure 5:**
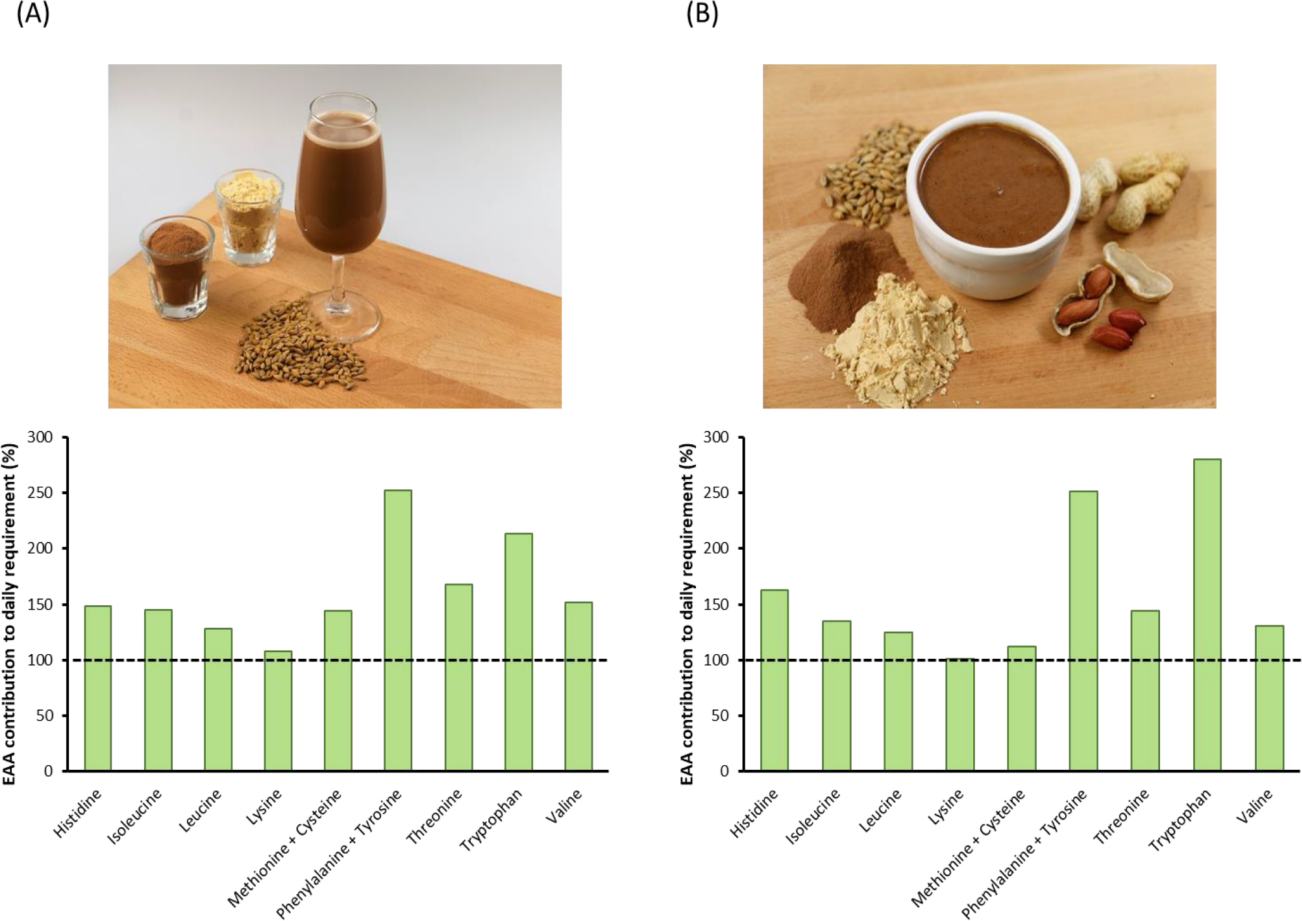
Product appearance and predicted contribution to daily essential amino acid requirement (%) of alternative therapeutic milk (A) and alternative ready-to-use therapeutic food (B) (unpublished data).

The African beer industry is growing, with revenue estimated to be USD 29.4 billion in 2023 and a predicted CAGR of 6.2% up to 2027.^124^ In 2020, BSG production lay in the range of 0.5–0.9 million tonnes with this figure expected to increase to 0.61–1.60 million tonnes in 2040, representing a CAGR of 7.71%.^125^ UNICEF, the largest purchaser of RUTF, continues to drive and facilitate local production in program countries in order to increase self-sufficiency and reduce costs associated with offshore production.^126^ Thus, the widespread availability of BSG across Africa could allow for the production of BSG-derived ingredients locally, reducing the need for the importation of components, such as milk powder. This represents a significant opportunity for the use of BSG-derived ingredients for the formulation of plant-based, lactose-free, and sustainable therapeutic foods to address the hunger crisis. In particular, the previously discussed high nutritional value and low environmental impact of the BSG protein isolate highlight the potential of this ingredient as a high-quality alternative protein source in therapeutic foods. Alternative TM and RUTF can be formulated through the replacement of milk powder with a combination of BSG protein isolate and pea protein to achieve a high-quality protein. Images of the alternative therapeutic foods along with their predicted contributions to the percentage of the daily requirement of each essential amino acid per gram of protein as outlined by the WHO are shown in Figure 5 (unpublished data). Complementation of BSG protein isolate with pea protein increased the score of the BSG limiting amino acid lysine (101–108%) while also resulting in the required values of the pea protein-deficient sulfur-containing amino acids methionine and cysteine being provided (112–144%). Each of the remaining indispensable amino acids had scores of ≥120%, highlighting the achievement of a complete essential amino acid profile using a plant-based protein blend. The predicted nutritional values of the alternative foods met the target nutritional profiles of F100 therapeutic milk and RUTF as outlined by the WHO.^118,127^ Compared to the commercially available products, the alternative formulations were predicted to be similar in terms of calorie content with slightly higher fat and carbohydrate contents and comparable or higher levels of protein (Table 5, unpublished data).

**Table 5:** Predicted Nutritional Composition of Alternative Therapeutic Foods in Comparison to Standard Therapeutic Foods and Formulation Guidelines

	therapeutic milk (TM) (g/100 mL)			RUTF (g/100 kcal)		
	requirement^a^	standard TM	alternative TM	requirement^a^	standard RUTF	alternative RUTF
energy (kcal)	95–105	100	102	520–550	543	548
fat	4.9–6.9	5.8	6.6	5–7	6.1	6.2
% energy from fat	45–60	53.0	58.2	45–60	50.2	55.7
carbohydrates	7–12	9.0	10.9	b	6.6	7.8
% energy from carbohydrates	28–45	36.0	42.8	b	36	31.1
protein	2.3–3.1	3.0	3.0	2.5–3.0	2.6	3.0
% energy from protein	10–12	11.0	11.7	10–12	10.2	12.0

a Requirements for therapeutic milk and RUTF were outlined by the WHO.

b Required value not specified.

Aside from TM and RUTF, BSG-derived ingredients could also supplement and enhance the nutritional value of other specialized foods distributed by humanitarian aid agencies. Fortified blended foods (FBFs) are blends of milled cereals and legumes fortified with vitamins and minerals which are usually mixed with water and cooked as a porridge.^128^ Corn–soy blend (CSB) is the most commonly used FBF, although wheat–soy blend (WSB) may also be used, with some formulations containing vegetable oil, milk powder, or whey protein concentrate.^129^ The USAID has encouraged the development of alternative FBFs, with blends of sorghum and cowpea mainly investigated due to their complementary amino acid profiles and ability to thrive in harsh conditions (drought, waterlogging).^130^ Studies have shown that FBFs formulated with alternative cereals and legumes are not inferior to CSB in terms of anemia risk and height and weight gain,^131,132^ indicating that the use of alternative protein sources in FBFs is indeed viable. The lack of available information in the literature indicates that the application of BSG in the FBF formulation has yet to be investigated. Hence, the use of BSG or BSG-derived ingredients provides a unique opportunity to improve the protein quantity and quality of FBFs through replacement or supplementation of the currently used cereal and legume sources. High-energy biscuits (HEBs) and compressed food bars are two other examples of food aid products containing wheat flour as their main ingredient. Considering the ability of the protein-rich and fiber-rich BSG-derived ingredients to enhance the nutritional and technological characteristics of bread and pasta through partial flour replacement,^83,86^ similar benefits could be achieved with the inclusion of these ingredients in HEBs and compressed food bars. BSG-derived fiber-rich and protein-rich ingredients could also be used as supplementary powders to enhance the protein and/or fiber content of foods, similar to the single-serve micronutrient powder or “sprinkles” sachets currently provided by the World Food Programme.^133^

## Upcycled BSG and the Developed World

Malnutrition is not confined to undernourishment in the developing world, with the 2021 Global Nutrition Report stating that poor diet was the leading cause of 12 million avoidable deaths globally in 2018, a growth of 15% since 2010.^134^ The prevalence of obesity is rising worldwide, with more than 1.9 billion adults considered overweight in 2016, and an estimated 379 million children and adolescents affected by overweight or obesity from 2016 to 2020.^135^ The dietary fiber gap is also a continuing concern, with an estimated 95% of US children and adults not consuming the recommended 25–30 g of fiber per day,^136^ while daily fiber intake in EU adults ranges from 16 to 24 g.^137^ Schools are well-positioned to provide nutrition education and promote healthy eating through school feeding programmes such as the USDA National School Lunch Program and School Breakfast Program.^138,139^ Studies have shown that food selection patterns among students display preferences for products such as pizza, cookies, and chips^140,141^ even when healthier alternatives are available. One option to alleviate this is to increase the nutritional value of staple foods such as bread and pasta and provide healthier alternatives to foods such as muffins, biscuits, and pizza, which are unlikely to be eliminated from the diet completely. The application of protein-rich and fiber-rich BSG-derived ingredients in such foods was recently investigated and the nutritional value compared to standard commercially available products (Table 6) (unpublished data). Each alternative product has significantly higher dietary fiber contents than the commercial foods, particularly the sponge cake, crackers, and pizza crust which contain 12-fold, 8.7-fold, and 4-fold more dietary fiber, respectively. Moreover, the products are ones with which children and adolescents are already familiar, reducing the risk of negative perception and reluctance to try, barriers which are often associated with the introduction of novel foods into the diet.^142^ An opportunity also exists for the provision of such foods to other cohorts, for example, the aging population, with an ongoing shift in demographics indicating that the global population of individuals aged 60 years and above is expected to double to 2.1 billion between 2020 and 2050.^143^ Maintaining health, independence, and quality of life are priorities for the aging population;^144^ however, the prevention of age-related disability can be jeopardized by the involuntary and progressive loss of muscle mass and function, a condition known as sarcopenia.^145^ It is estimated that up to 46% of older adults do not meet the recommended protein intake level of 0.8 g/kg/day,^146^ with research suggesting that the optimal protein intake for adults older than 65 years is in fact closer to at least 1.0–1.2 g/kg/day.^147,148^ The inclusion of BSG-derived ingredients has the potential to increase the protein content of foods by up to 2.8 times that of standard products (Table 6). Furthermore, such food types are attractive for fortification, with a recent study citing bread, pasta, cakes, and biscuits as preferred products by older consumers for protein fortification.^149^ The increased dietary fiber content of the foods may also be beneficial for this cohort, with studies highlighting the potential association of fiber with improved cognitive function, mitigation of sarcopenia, and better physical performance.^150−152^

**Table 6:** Predicted Nutritional Composition of Alternative Food Products Containing BSG-Derived Ingredients in Comparison with Standard Commercial Products^a^

	protein-rich BSG-derived ingredient (% based on flour)	fiber-rich BSG-derived ingredient (% based on flour)	energy (kcal)	protein (g)	carbohydrates (g)	dietary fiber (g)	fat (g)
**bread**							
standard			274	10.7	47.5	4.0	4.5
alternative	13.0	5.0	254	13.0	47.0	6.1	4.0
**pasta**							
standard			157	5.8	30.7	1.8	0.9
alternative	15.0	9.0	227	10.6	41.0	8.0	2.3
**muffin**							
standard			372	6.9	45.7	1.8	18
alternative	30.5	12.0	475	9.1	50.3	6.1	28.5
**sponge cake**							
standard			290	5.4	61.0	0.5	2.7
alternative	40.0		292	15.0	44.8	6.0	8.8
**pizza crust**							
standard			262	6.2	47.7	1.5	3.9
alternative	13.0	5.0	260	13.0	44.9	6.2	5.8
**biscuit**							
standard			450	5.0	75.0	0.0	15.0
alternative		18.0	454	7.4	74.8	6.1	18.9
**cracker**							
standard			500	6.3	62.5	1.0	28.1
alternative	13.0	5.0	346	14.6	62.9	8.7	7.5

a Nutritional composition of standard products obtained from USDA FoodData Central. Data expressed per 100 g of product.

## Opportunities and Challenges for the Utilization of Upcycled BSG

Despite the increasing interest in food waste valorization and the creation of value-added products, upcycled food remains a relatively novel concept which faces several challenges but also many opportunities. A significant opportunity for upcycled food manufacturers is the rapid growth of the global upcycled food market, standing at USD 46.7 billion in 2019 with a projected growth of 5% year-on-year for the next ten years. North America currently leads the global market for upcycled ingredients with a market share of 48%, followed by Asia Pacific (22.6%), Europe (21.6%), and Latin America (5.6%).^153^ Increasing consumer awareness regarding food waste and sustainable food production is a significant driver of growth in the upcycled food market, and with the number of food and beverage products containing upcycled ingredients increasing by 122% in 2021, it is evident that food and beverage companies are taking steps to meet this increased demand. Despite this, there are still cohorts of consumers who are unaware of what upcycled products are. A positive step toward consumer education was the development of “Upcycled Certified”, the world’s first third-party certification program for upcycled products and ingredients. Developed by the Upcycled Food Association, the program certifies ingredients that contain at least 95% upcycled inputs by weight (upcycled ingredient; UI), products that contain a minimum of 10% upcycled inputs by weight (product containing upcycled ingredient; PUI), and products containing less than 10% upcycled inputs by weight (minimal content PUIs). Use of the Upcycled Certified logo allows clear communication to consumers regarding the presence of upcycled ingredients in their food products, with a study showing that more than 50% of consumers had increased intent to purchase Upcycled Certified foods when the logo was visible on the packaging.^154^

One challenge facing the widespread utilization of upcycled BSG is the batch variability of the raw material in terms of chemical composition. While this may not be an issue for large industrial breweries, notable variations in composition have been reported for BSG samples obtained from microbreweries, potentially hindering large-scale implementation of a standardized process and achieving a consistent end product.^155^ Waste stream inconsistency will in turn affect the ingredient/product quality and, subsequently, consumer acceptability of upcycled foods. The commercial success of upcycled BSG is highly dependent on consumer acceptance, with consumers often hesitant to support concepts with which they are not familiar.^156^ However, studies have shown that although consumer knowledge of upcycled foods is low, there is a willingness to purchase such products once informed.^157−159^ Consumer sociodemographic characteristics reportedly influence the acceptability of upcycled foods, with younger consumers and consumers with a high-income level and high level of education more inclined to choose upcycled foods.^157,160,161^ The development of upcycled foods with sensory attributes comparable to those of conventional products is a significant challenge for manufacturers, with the adverse effects of BSG inclusion on food sensory attributes already being well-documented and discussed. However, the use of BSG-derived ingredients is likely to improve the technological and sensory qualities of foods compared to the use of native BSG, with evidence of this already observed in bread and pasta.^83,86,91^ Moreover, the way in which upcycled BSG products are framed to the consumer is important, with a study by Stelick et al. reporting that although a BSG-containing cereal bar was outperformed by the control bar in terms of hedonic measurements, a significant positive effect on purchase intent was observed when the participants were informed about the nutritional and sustainability aspects of the product.^162^ However, it is important that consumers are not misconceived into presuming that all upcycled foods are inherently more nutritious and sustainable than standard products; thus, such claims should be backed by sufficient evidence.^99^ Upcycled food manufacturers should also be aware of the significant impact that price has on consumer acceptability, with a willingness to purchase often dependent on marketing communication strategies. While some consumers may be willing to pay a premium price for upcycled products which are marketed as nutritious and environmentally friendly,^158,162^ upcycled foods are often negatively perceived as containing “waste” material for which consumers expect to pay a lower price.^99,156,159,163,164^ This was observed in the case of the BSG-containing cereal bar, whereby the optimal pricing point was determined to be lower than the control bar.^162^ However, a separate study found that consumer attitude toward a higher price point improved when informed about the often-higher production costs associated with upcycled foods, an encouraging find.^159^

Upcycled foods will likely face regulatory challenges going forward, particularly those which fall into the “novel foods” category. Novel foods are those which were not produced or used for human consumption in the European Union (EU) before 15th May 1997 and require premarket authorization (EU Regulation 2015/2283).^165^ BSG in its native form, which undergoes minimal processing (drying, milling), is not considered a novel food as it has a history of consumption within the EU prior to 1997. Moreover, a recent consultation request has deemed protein-rich (BSG-P, 50% protein) and fiber-rich (BSG-F, 70% fiber) BSG-derived ingredients as not novel, as the fractions are obtained by a mechanical process which does not result in any chemical changes in its constituents.^166^ The classification of these ingredients as not novel is beneficial, as it eliminates the requirement for authorization and simplifies their entrance to the market. On the other hand, barley rice protein isolate derived from BSG is considered a novel food, as it has no history of use in the EU and the composition is significantly different from that of native BSG. Rahikainen et al. outlined the process of obtaining authorization as a novel food as a demanding one which may be hindering the transition to sustainable foods, with the authors suggesting that the novel food status of all major alternative proteins should be clarified by the European Food Safety Authority (EFSA) without request, thus eliminating the requirement to file an initial application for consultation and speeding up the approval process.^167^ Not only is the process time-consuming, but it can often be costly, a considerable hurdle to many start-up companies. However, despite the demanding nature of the process, the classification of a product as a novel food also presents an opportunity for companies, allowing for data protection and individual authorization for five years for placing on the market the novel food.^165^ In the US, the Food and Drug Administration (FDA) allows self-certification that ingredients or products are “generally recognized as safe” (GRAS) through scientific evidence of safety or evidence of a history of consumption of the substance in food prior to 1958, often a quicker process than EU novel food authorization.

Food safety aspects relating to the utilization of upcycled BSG as a food ingredient should also be considered. Fungal species such as *Fusarium*, *Penicillium*, *Alternaria*, and *Rhizopus* have been detected in barley grains, with favorable conditions during germination and kilning (moisture, nutrient availability, aeration, humidity) increasing the risk of mycelial growth and mycotoxin production.^168,169^ Although the drying phase of the malting process inhibits fungal proliferation, mycotoxin synthesis continues throughout the brewing process, with the occurrence of aflatoxins, trichothecenes, fumonisins, ochratoxin A, and zearalenone in malted barley, BSG, and final beer products reported in the literature.^169,170^ Moreover, studies have shown that some mycotoxins may adsorb to the grain during brewing,^171,172^ highlighting the risk of obtaining a contaminated byproduct. In addition, as the high moisture content of BSG makes it susceptible to spoilage postharvest, any delay in processing the byproduct poses an additional risk of further microbial growth and toxin production. Thus, it is imperative that the presence of microbial contamination and mycotoxins in upcycled BSG ingredients is actively monitored to ensure a safe product for the consumer.

To conclude, the use of BSG, a plentiful, nutritious byproduct, as feed or waste is no longer feasible given the ongoing global food crisis and the increasing pressure on our natural resources. Hence, efforts to upcycle BSG and produce value-added ingredients are of great interest from both economic and environmental points of view. Several strategies are available for the upcycling of BSG, including enzymatic hydrolysis, fermentation, and ingredient fractionation. Comparison of the technofunctional properties of BSG protein isolate to standard plant protein isolates highlights its suitability for application in various food matrices, while the efficacy of protein-rich and fiber-rich BSG-derived ingredients for the fortification of bread and pasta has been demonstrated. BSG-derived ingredients have the potential to play a major role in efforts to address and reduce world hunger, with BSG protein isolate demonstrating its function as a high-quality protein source in the development of lactose-free, sustainable, and therapeutic foods. The future of BSG-derived ingredients in the Western world is also evident, with the potential to increase the nutritional value of staple foods such as bread and pasta and other products including sponge cake, crackers, biscuits, and pizza bases, with the aim of reducing the risk of dietary-related disease. Upcycled BSG may face some challenges with regard to regulatory status and gaining consumer acceptance; however, the upcycled food market is experiencing rapid growth which is projected to continue, highlighting increasing consumer awareness and acceptance. Of importance are the communication strategies used by manufacturers, which should take into consideration consumer sociodemographic characteristics and the requirement for clear, transparent communication about the potential benefits of upcycled BSG products. Overall, the future of upcycled BSG as a sustainable and nutritious ingredient to address world hunger and malnutrition is a promising one.

## Abbreviations

BSG: Brewers’ spent grain
SDG: sustainable development goal
FLW: food loss and waste
WHO: World Health Organization
FAO: Food and Agriculture Organization of the United Nations
DM: dry matter
FODMAP: fermentable oligo-, di-, and monosaccharides and polyols
EAA: essential amino acids
NEAA: nonessential amino acids
LAB: lactic acid bacteria
SF: source of fiber
HF: high fiber
PPI: pea protein isolate
SPI: soy protein isolate
LPI: lentil protein isolate
FBPI: faba bean protein isolate
WLPI: white lupin protein isolate
BLPI: blue lupin protein isolate
LCA: life cycle assessment
US: United States
TM: therapeutic milk
RUTF: ready-to-use therapeutic food
PDCAAS: Protein digestibility corrected amino acid score
FBF: fortified blended food
HEB: high-energy biscuits
CSB: corn–soy blend
WSB: wheat–soy blend
FDA: Food and Drug Administration’
GRAS: generally regarded as safe
EFSA: European Food Safety Authority
EU: European Union
